# Surgical Level Selection in Checkrein Syndrome: A Pathology-Oriented Approach to Flexor Hallucis Longus Lengthening

**DOI:** 10.7759/cureus.110810

**Published:** 2026-06-14

**Authors:** Yoichi Toyoshima, Haruka Emori, Seiichi Niikura, Yuichiro Okazaki, Hirotaka Kato, Mayuko Noda, Yoshifumi Kudo

**Affiliations:** 1 Orthopedics Surgery, Tokyo Metropolitan Ohtsuka Hospital, Tokyo, JPN; 2 Orthopedics Surgery, Showa Medical University School of Medicine, Tokyo, JPN

**Keywords:** checkrein syndrome, foot deformity, knot of henry, surgical decision-making, tendon lengthening

## Abstract

Checkrein syndrome presents as a dynamic flexion deformity of the hallux that varies with ankle position and may occur after trauma. Surgical management has traditionally focused on proximal intervention targeting the flexor hallucis longus; however, inconsistent outcomes and recurrence indicate that a single-mechanism explanation may be insufficient.

This narrative review proposes a pathology-oriented framework for surgical level selection based on a dual-mechanism concept distinguishing proximal tethering from distal intertendinous coupling at the knot of Henry. Within this framework, deformity patterns are interpreted in relation to both the origin and transmission of pathological tension, including propagation to the lesser toes via distal coupling.

Surgical level selection should therefore be guided by the underlying pathological mechanism rather than a uniform strategy. A pathology-oriented approach based on deformity pattern and dynamic findings offers a practical basis for optimizing surgical outcomes and reducing recurrence.

## Introduction and background

Checkrein syndrome presents as a dynamic flexion deformity of the hallux that varies with ankle position, most commonly after lower limb trauma. The term “checkrein” is derived from the strap used to limit a horse’s head movement and reflects the characteristic phenomenon in which ankle dorsiflexion increases tension within the flexor system, resulting in accentuation of toe flexion deformity. The condition is classically attributed to impaired tendon gliding of the flexor hallucis longus (FHL), typically due to adhesion or tethering at the fracture site or within surrounding scar tissue [1-3]. Several case reports and small series have described similar post-traumatic mechanisms of FHL tethering [4-6]. Based on this proximal tethering concept, surgical management has traditionally focused on interventions at the hindfoot or leg, including adhesion release and tendon lengthening at the fracture site, retromalleolar region, or tarsal tunnel [2,3,7]. Although these approaches generally improve symptoms, recurrence and incomplete correction have been reported, suggesting that proximal pathology alone does not fully explain all clinical presentations [1,2]. In contrast, distal procedures performed at the midfoot or plantar region -- particularly at the knot of Henry -- have also demonstrated favorable outcomes, often with simpler surgical exposure and lower recurrence rates [7,8].

Proximal tethering explains the generation of tension but does not fully account for its propagation. The FHL and flexor digitorum longus (FDL) are connected at the knot of Henry, allowing force to be transmitted to the lesser toes and providing a basis for distal coupling [8]. Clinical reports describing multi-toe involvement further support the concept that pathological tension may propagate through this intertendinous network rather than remaining localized [9,10]. 

Despite increasing recognition of these mechanisms, no clear consensus exists regarding the optimal level of surgical intervention. In clinical practice, surgical decision-making remains largely experience-based, with limited guidance on how to select between proximal and distal approaches [1,7,8]. The purpose of this narrative review is to evaluate the clinical relevance of surgical level selection in the treatment of checkrein syndrome by comparing proximal and distal strategies. The proposed classification and pathology-oriented framework are intended as conceptual tools derived from the available literature and have not yet been clinically validated.

Methods

We conducted a focused narrative review to examine the clinical relevance of surgical level selection for FHL tendon lengthening in checkrein syndrome. A targeted literature search was performed using PubMed, employing combinations of the following keywords: “checkrein syndrome”, “flexor hallucis longus”, “knot of Henry”, and “toe deformity.” Additional relevant articles were identified through manual screening of reference lists.

Eligible studies included review articles, case series, and case reports describing the pathophysiology, anatomical characteristics, and surgical management of checkrein syndrome. Particular emphasis was placed on studies that provided insight into the level of surgical intervention, including proximal approaches (fracture site, retro-malleolar region, and tarsal tunnel) and distal approaches (midfoot and plantar region, including the knot of Henry).

Rather than applying strict inclusion and exclusion criteria, studies were selected based on their relevance to the central clinical question: determining the optimal level of intervention according to the underlying pathological mechanism. Data were qualitatively synthesized to examine deformity generation, force transmission, and surgical outcomes according to intervention level, forming the basis for a pathology-oriented framework for surgical decision-making.

Literature published through January 2026 was considered for inclusion. Studies were screened for relevance to the objectives of this review by evaluating titles, abstracts, and full texts when necessary, with particular attention to pathophysiology, anatomical characteristics, and surgical management relevant to surgical level selection in checkrein syndrome. A total of 45 articles were included in the final qualitative synthesis. Because this study was designed as a focused narrative review rather than a systematic review, formal risk-of-bias assessment and quantitative evidence grading were not performed.

## Review

In this review, the key clinical issue is not the technique of tendon lengthening itself, but the selection of the appropriate level of intervention according to the underlying pathological mechanism. Checkrein syndrome has traditionally been explained by proximal tethering of the FHL, which accounts for the dynamic relationship between ankle motion and hallux deformity [1-3]. However, variability in clinical presentation, including multi-toe involvement and inconsistent outcomes following proximal procedures, suggests that this explanation is incomplete [2,11,12]. The following sections examine the mechanisms of deformity generation and transmission and their implications for clinical decision-making.

Pathophysiology of checkrein syndrome

Checkrein syndrome is most commonly understood as a dynamic flexion deformity of the hallux resulting from mechanical restriction of the FHL tendon. This reflects the “constant length phenomenon,” in which tethering fixes tendon excursion during ankle motion [13]. The deformity typically worsens with ankle dorsiflexion and improves with plantarflexion, reflecting a length-dependent limitation of tendon excursion [14].

This restriction most frequently arises from post-traumatic adhesion or entrapment of the FHL within anatomically constrained regions such as the deep posterior compartment or retromalleolar tunnel [1,2]. Several case reports and small series have described similar post-traumatic mechanisms of FHL tethering [4,5,6]. The tendon may become incorporated into callus or scar tissue, impairing normal gliding. Direct intraoperative evidence supports this mechanism. Entrapment of the FHL within fracture fragments, particularly in talar injuries, has been associated with immediate restoration of hallux motion following surgical release, indicating a purely mechanical obstruction [15]. Similar findings have been reported in tibial shaft fractures, where fibrous adhesions extending from the fracture site to the musculotendinous unit restrict tendon mobility [11]. Temporal patterns further support a post-traumatic fibrotic process. In clinical series, deformity often develops several months after the initial injury rather than acutely, suggesting progressive scar maturation as a key contributor [2].

During ankle dorsiflexion, increased excursion of the FHL is required; when this excursion is restricted, compensatory flexion occurs at the interphalangeal joint of the hallux. Plantarflexion reduces this tension and partially restores alignment, producing the characteristic dynamic deformity [2,14]. While this proximal mechanism explains the generation of deformity in many cases, it does not fully account for all clinical presentations. In particular, involvement of the lesser toes, variability in deformity severity, and inconsistent surgical outcomes suggest that additional factors contribute to the overall pathophysiology [2,11,12].

Evidence From Trauma Cases

The association between checkrein syndrome and trauma is consistently observed, with most cases arising after fractures of the tibia, fibula, ankle, or hindfoot. These injuries promote adhesion formation and restrict FHL excursion, a mechanism directly confirmed by intraoperative findings. In ankle fractures, adhesion of the FHL within the deep posterior compartment can impair tendon gliding, with immediate restoration of motion following surgical release [16], while similar findings have been reported in tibial shaft fractures complicated by tibiofibular synostosis, where the tendon is constrained by both osseous and fibrous structures extending proximally [11].

Entrapment of the FHL within fracture-related structures has also been demonstrated in hindfoot injuries, including talar fractures, where mechanical obstruction is confirmed by immediate correction after release [15]. Additional reports describe involvement of both the FHL and FDL, suggesting that adhesion may affect multiple components of the flexor system [12,14]. Clinical series further indicate that deformity often develops several months after injury, reflecting progressive fibrosis rather than acute entrapment [3,11], with comparable mechanisms described across multiple case reports involving tibial and ankle fractures [17,18]. These observations support post-traumatic adhesion and proximal tethering as important mechanisms of deformity generation. Nevertheless, variability in tendon involvement and frequent extension to the lesser toes suggest that the effects of proximal pathology are not always confined to a single anatomical site, supporting a potential role for distal force transmission.

Limitations of the Proximal-Only Model

Despite substantial evidence supporting proximal tethering of the FHL as a primary mechanism, several limitations of this model have become increasingly apparent. The deep posterior compartment, where the FHL courses alongside neurovascular structures, is anatomically constrained and susceptible to increased pressure and restricted tendon excursion, providing a structural basis for tendon dysfunction [19].

First, clinical outcomes following proximal intervention are inconsistent. In a representative series, recurrence was observed in patients undergoing adhesion release and Z-lengthening at the fracture site, whereas no recurrence occurred in those treated with midfoot procedures [3]. Similar discrepancies have been reported in case-based studies, where proximal procedures resulted in residual deformity, while distal lengthening achieved more reliable correction [12]. These findings indicate that proximal release alone may be insufficient. Second, precise localization of the pathological site remains challenging. In several reports, including those by Lee and Rosenberg, the site of tendon tethering could not be reliably identified preoperatively, and intraoperative findings sometimes differed from preoperative assumptions [2,14]. This diagnostic uncertainty limits the predictability of proximal interventions and raises the possibility that pathology may extend beyond the clinically apparent site of restriction. Third, certain deformity patterns are not adequately explained by isolated proximal tethering. Involvement of multiple lesser toes has been documented in several series, indicating that deforming forces are not confined to the hallux [2,11]. While this phenomenon has been attributed in part to intertendinous connections between the FHL and FDL, the proximal model alone does not account for the coordinated and pattern-dependent nature of multi-toe deformity. Fourth, alternative mechanisms have been proposed that extend beyond simple mechanical tethering. Subclinical compartment syndrome and ischemic contracture of the deep posterior compartment have been reported in association with checkrein-like deformities, suggesting that tendon dysfunction may arise from broader pathophysiological processes [1,16]. Additional reports describing Volkmann-type contracture and compartment-related pathology further support the existence of non-localized mechanisms affecting tendon behavior [20,21]. Missed or subclinical compartment syndrome has also been implicated as a contributor to tendon dysfunction in post-traumatic cases [22].

Such limitations suggest that checkrein syndrome cannot be fully explained by a single proximal mechanism. While proximal tethering represents a critical source of pathological tension, it does not adequately account for variability in deformity pattern, the extent of tendon involvement, or surgical outcomes. These inconsistencies suggest that additional mechanisms -- particularly those related to distal force transmission through intertendinous connections -- warrant consideration.

Anatomical basis of distal coupling

Anatomy of the Knot of Henry

The knot of Henry represents the anatomical intersection between the FHL and FDL tendons in the plantar midfoot. At this level, the FHL courses obliquely beneath the FDL and gives rise to tendinous slips that connect with the digital tendons, forming a functional bridge between the hallux and lesser toes [23,24].

This region functions as an integrated tendon interface. The quadratus plantae contributes to this integration by inserting not only into the FDL but also into intertendinous slips, creating a multilayered system in which extrinsic and intrinsic components interact [25,26]. Cadaveric studies consistently demonstrate that these interconnections are present in the vast majority of specimens, indicating that they represent a fundamental anatomical feature rather than a rare variation [26].

Although the precise morphology varies, the presence of at least one communicating slip from the FHL to the FDL is the dominant configuration. Bidirectional or absent connections are less common but have been documented, reflecting a spectrum of anatomical organization [27,28]. This structural arrangement provides the anatomical basis for distal transmission of force and establishes the substrate for coupling between the hallux and lesser toes.

Variations of FHL-FDL Connection

Despite its high prevalence, the configuration of FHL-FDL interconnection demonstrates substantial variability, which has direct functional and surgical implications.

Several anatomical studies have demonstrated substantial variability in the configuration of the FHL-FDL connection. The most common pattern consists of a unidirectional slip from the FHL to the FDL, although double-slip, multi-slip, and absent communication patterns have also been reported [23,29,30]. Contributions from the FHL are most frequently directed to the second and third toes, with occasional extension to the fourth digit [25,29]. Functional studies further demonstrate that traction on the FHL can produce coordinated flexion of both the hallux and lesser toes, depending on the configuration [24].

These variations influence force transmission across the forefoot and the functional effects of surgical intervention.

Functional Significance

The anatomical interconnection at the knot of Henry has important functional implications for coordinated toe motion and load distribution.

Quantitative studies show that the FHL contributes substantially to flexion of the second and third toes, accounting for a significant proportion of tendon cross-sectional area in these digits [29], supporting its role as part of a multi-digit flexor system rather than an isolated hallux flexor. Biomechanically, this configuration facilitates force redistribution during gait, particularly at toe-off, contributing to propulsion and forefoot stability [25], while cadaveric studies demonstrate that traction on the FHL produces coordinated flexion across multiple toes, consistent with an integrated tendon system [24]. Imaging studies corroborate these findings, demonstrating consistent contribution of the FHL to the lesser toes and confirming distal force transmission [31]. The knot of Henry has also been identified as a region of increased mechanical stress, where repetitive tendon interaction may contribute to pathology [32].

The level of tendon division determines whether force transmission is preserved or interrupted. Procedures performed proximal to the knot may allow continued transmission through intact interconnections, whereas distal division more directly alters multi-toe mechanics [32,33].

This distal coupling explains the occurrence of multi-toe deformity in checkrein syndrome, in which proximal tension is transmitted through the intertendinous network. The quadratus plantae may further modulate this process, as its insertion into the FDL can redistribute tension across the digital tendons, potentially amplifying multi-toe involvement in the presence of strong intertendinous coupling [25,26].

Biomechanical Integration of Distal Coupling

The functional role of the knot of Henry can be understood as a load-dependent coupling system. Through the interconnection between the FHL and FDL, tension generated proximally can be transmitted to the lesser toes, providing a pathway for propagation of deforming forces.

This mechanism explains how restriction of FHL excursion may result in multi-toe deformity. Variability in deformity pattern reflects differences in force distribution through this intertendinous network, linking anatomical structure to clinical presentation [24,34].

Clinical presentation patterns

Hallux-dominant deformity in checkrein syndrome presents as isolated flexion of the hallux that varies with ankle position, typically worsening with dorsiflexion and improving with plantarflexion [2,14]. This finding reflects restricted excursion of the FHL without effective transmission of tension to the lesser toes. The deformity results from unopposed FHL action, producing interphalangeal flexion that becomes more pronounced during ankle dorsiflexion and toe-off, when the hallux bears load and contributes to propulsion [35,36]. Limited involvement of the lesser toes indicates minimal propagation of force through the FHL-FDL network.

**Figure 1:**
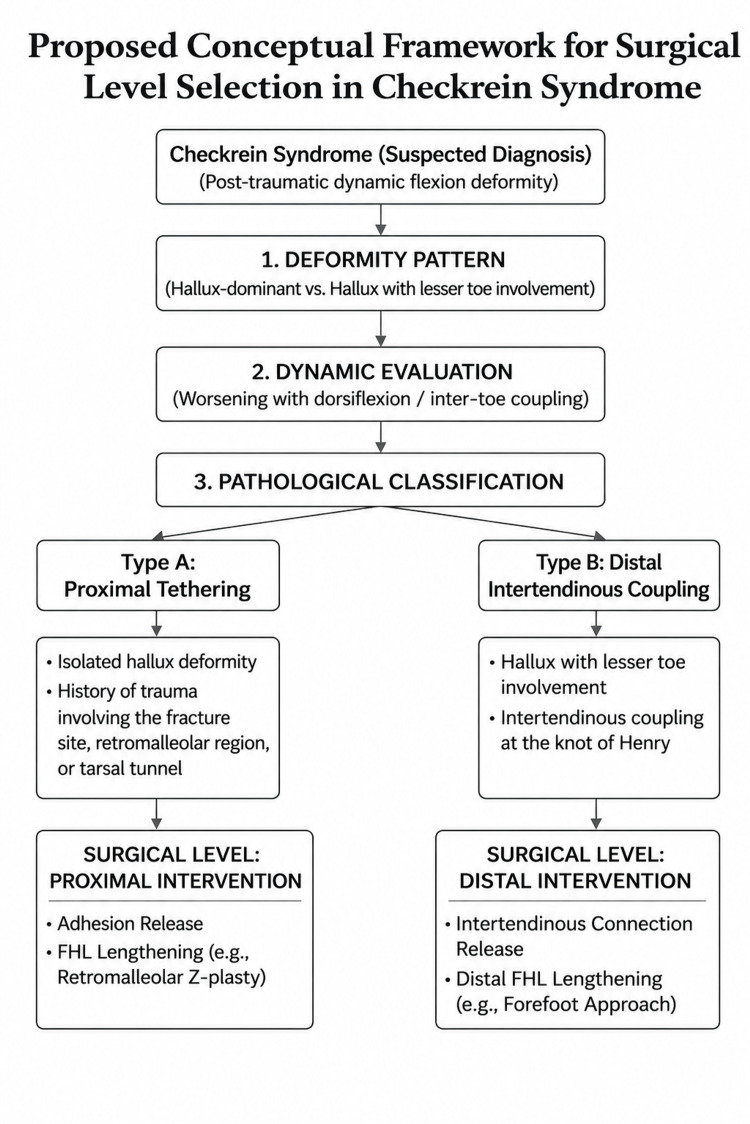
Proposed conceptual framework for surgical level selection in checkrein syndrome This proposed conceptual framework illustrates a pathology-oriented approach to surgical level selection based on the dominant pathological mechanism. Hallux-dominant deformity without lesser toe involvement is associated with proximal pathology (Type A: FHL tethering at the fracture site, retromalleolar region, or tarsal tunnel), for which proximal interventions such as retromalleolar Z-plasty or fracture-site release are indicated. In contrast, deformity involving the hallux and lesser toes, particularly with synchronized flexion, may indicate distal intertendinous coupling at the knot of Henry (Type B), supporting consideration of distal procedures such as intertendinous release or midfoot/forefoot FHL lengthening. Careful clinical and dynamic assessment is essential for accurate pathological classification and appropriate surgical level selection. The framework is intended as a hypothesis-generating clinical aid derived from available anatomical, biomechanical, and clinical observations and has not yet been prospectively validated. This figure was created by the authors using Microsoft PowerPoint (Microsoft Corp., Redmond, WA, USA) and is original without the use of generative artificial intelligence tools.

This presentation is consistent with a proximal-dominant mechanism and serves as a clinical indicator of localized FHL tethering within the classification framework illustrated in Figure 1. Comparable deformity patterns have been described in claw and hammer toe conditions, where imbalance between intrinsic and extrinsic musculature leads to interphalangeal flexion [37].

Multi-toe Deformity

Multi-toe deformity in checkrein syndrome represents a distinct clinical pattern characterized by simultaneous involvement of the hallux and one or more lesser toes, typically exacerbated by ankle dorsiflexion. Unlike isolated forefoot deformities, this deformity reflects propagation of pathological tension from the FHL through intertendinous connections to the lesser toes [2,11]. This coordinated deformity pattern provides a key clinical indicator of distal force transmission rather than isolated proximal tethering. In contrast to general toe deformities, the multi-toe pattern in checkrein syndrome reflects dynamic propagation of tension through the FHL-FDL network [35,36,38]. These findings directly support the classification framework presented in Figure 1, in which hallux-dominant deformity reflects localized proximal pathology, whereas multi-toe involvement indicates transmission-driven deformity mediated by intertendinous coupling.

Clinical observations further support that a single-tendon pathology can produce multi-digit deformity. For example, contracture of the FHL alone has been reported to induce clawing of both the hallux and lesser toes, with preservation of some hallux function through interconnection with the FDL [39]. From a clinical perspective, multi-toe involvement is associated with increased symptom severity. Altered toe mechanics lead to redistribution of plantar pressure, resulting in metatarsalgia, callosities, and metatarsophalangeal joint instability [36]. Progressive structural changes, including plantar plate insufficiency, may further exacerbate deformity and forefoot dysfunction [40].

This phenotype supports the concept of distal coupling. The coordinated involvement of multiple toes suggests that deforming forces are transmitted through intertendinous connections rather than arising independently in each digit. This interpretation is consistent with reports describing multi-digit deformity following localized injury or tendon dysfunction [9,10]. Clinically, multi-toe deformity is often more rigid and less responsive to conservative treatment, frequently requiring surgical intervention addressing both soft tissue imbalance and structural alignment. From a mechanistic perspective, this deformity represents a transmission-dominant phenotype, in which distal intertendinous coupling plays a central role in deformity expression.

Dynamic Behavior

In checkrein syndrome, deformity is characteristically dynamic, varying with ankle position and loading conditions. Hallux flexion typically worsens with dorsiflexion and improves with plantarflexion, reflecting the tension-dependent behavior of the FHL [3,14]. Deformity is often accentuated during weight-bearing and toe-off, when flexor tension increases. Flexible deformities may be subtle at rest but become evident under loading, whereas fixed deformities persist regardless of ankle position [35,36].

These dynamic features provide important clinical cues for identifying the dominant mechanism and support classification within the framework illustrated in Appendix A.

Surgical strategies

Surgical strategy in checkrein syndrome is determined by the level of intervention rather than the specific technique. Proximal procedures target the source of pathological tension, whereas distal procedures modify its transmission through intertendinous connections [41]. Selection of the appropriate level depends on identifying the dominant mechanical driver of deformity. This relationship between deformity pattern and surgical level is summarized in Appendix A.

Proximal Interventions

Proximal surgical strategies have traditionally been regarded as the standard approach for checkrein deformity, targeting tethering or entrapment of the FHL in the posterior ankle or leg. Adhesion or synostosis at the fracture site can directly restrict tendon excursion, resulting in progressive clawing deformity [11]. Similarly, post-traumatic fibrosis associated with fracture callus or soft tissue injury impairs tendon glide and produces a dynamic deformity pattern [16]. These findings establish proximal tethering as a primary source of pathological tension.

Based on this concept, surgical techniques have focused on adhesiolysis and tendon lengthening. Z-plasty at the tarsal tunnel or retromalleolar region has demonstrated favorable clinical outcomes, including improvement in pain, range of motion, and function [2,7]. This is consistent with the “fixed-length phenomenon,” in which restriction of tendon excursion produces dynamic clawing that varies with ankle position [42]. In cases where pathology is localized to the proximal tendon course, such interventions can provide effective correction.

However, several limitations must be recognized. First, the pathological site is often multifactorial and not confined to a single proximal lesion. Adhesion, muscle contracture, and interosseous membrane involvement may coexist, limiting the effectiveness of isolated proximal release [16]. Second, procedures performed proximal to the knot of Henry cannot address distal pathology or intertendinous interactions, potentially resulting in incomplete correction [13]. Third, recurrence has been reported when the mechanical pathway of deformity is not fully addressed, particularly in the presence of distal coupling [2,12].

Proximal surgery often requires dissection through scarred or previously operated tissues, increasing technical complexity and the risk of neurovascular injury, especially in the tarsal tunnel region [2].

Distal Interventions

Distal surgical strategies have emerged as an important alternative, particularly in cases involving multiple toes or persistent deformity after proximal treatment. These techniques focus on the midfoot region or the knot of Henry, where the FHL and FDL intersect. Clinical studies have demonstrated that midfoot Z-plasty can achieve complete correction without recurrence, in contrast to proximal procedures [8]. Distal approaches also provide access to tendon segments in relatively unscarred tissue planes, facilitating reliable and reproducible surgical exposure [43]. Sanhudo also reported that distal lengthening offers technical simplicity, faster recovery, and favorable outcomes compared to proximal surgery [12]. More recently, combined procedures addressing both FHL and FDL at the knot of Henry have been proposed, directly targeting the intertendinous connection responsible for force transmission [44].

The level of tendon division is critical. Proximal release may reduce overall tension within the system, whereas distal intervention alters the balance of force transmission across digits. In certain configurations, inappropriate distal division may increase deformity of the lesser toes due to unopposed forces within the intertendinous network [13]. Understanding the anatomical and functional configuration of the knot of Henry is essential for surgical planning.

A key advantage of distal approaches is the ability to perform intraoperative dynamic assessment. Direct visualization under functional loading conditions allows precise adjustment of tendon length and tension, ensuring immediate correction of deformity [8]. This capacity for real-time evaluation may contribute to the lower recurrence rates reported in distal procedures. Overall, distal interventions provide a rational strategy for cases in which deformity reflects transmission of force rather than isolated proximal restriction, particularly in multi-toe involvement.

Comparative Interpretation

The relationship between deformity pattern and surgical level, as illustrated in Appendix A, integrates clinical presentation with the underlying mechanical pathway and provides a structured basis for selecting the appropriate intervention level. Proximal procedures primarily address the source of pathological tension, whereas distal procedures modify its transmission through intertendinous coupling. When both mechanisms coexist, treatment at a single level may be insufficient [3,7,8,12].

Pathology-oriented framework and clinical decision-making

In clinical practice, mixed-mechanism cases are not uncommon, particularly in patients with long-standing deformity or complex post-traumatic changes [2,11,16]. In such cases, both proximal tethering and distal intertendinous coupling contribute to the overall deformity pattern. Isolated intervention at a single level may fail to achieve complete correction if the secondary mechanism remains unaddressed.

A staged or combined approach should be considered. When proximal tethering is dominant but distal propagation is evident, initial proximal release may reduce the primary source of tension, followed by distal intervention if residual multi-toe deformity persists [2,12]. Conversely, in transmission-dominant patterns with suspected proximal contribution, distal procedures may be prioritized to address force distribution, with proximal release reserved for refractory cases [12,43]. This strategy is supported by the anatomical and biomechanical characteristics of the flexor system. The intertendinous connection between the FHL and FDL at the knot of Henry enables transmission of force to the lesser toes, providing a mechanism for propagation of deformity beyond the hallux [8,23,25,26,29,43]. Anatomical variation in the configuration and strength of these intertendinous connections may influence the extent of deformity propagation. Specimens with more robust or multiple tendinous slips have been shown to exhibit stronger functional coupling between the hallux and lesser toes, which may explain why multi-toe deformity occurs in some patients but not in others [23,29].

The deformity pattern depends on the relative contribution of proximal tethering and distal force transmission [2,7,8]. Deformity confined to the hallux and closely linked to ankle motion indicates proximal pathology, favoring intervention at the hindfoot or retromalleolar region. In contrast, multi-toe involvement with coordinated flexion reflects a transmission-driven pattern, supporting distal intervention at the midfoot or knot of Henry. Surgical decision-making is based on deformity pattern, dynamic findings, and underlying pathology. Initial assessment focuses on whether deformity is confined to the hallux or involves multiple toes, followed by evaluation of dynamic changes with ankle dorsiflexion, and culminates in identification of the dominant pathological mechanism. Imaging modalities such as ultrasonography may further support localization of pathology and refine surgical planning [45]. This stepwise evaluation provides a practical framework for aligning surgical level with the underlying pathological mechanism.

This framework also explains the mechanism of recurrence following proximal intervention. Release of proximal tethering restores tendon excursion but does not address distal force transmission through the FHL-FDL network. As a result, residual tension transmitted via the knot of Henry may persist, particularly in cases with strong intertendinous coupling, leading to incomplete correction or recurrence of multi-toe deformity [2,8,12].

Intraoperative dynamic assessment plays a critical role in these scenarios. Real-time evaluation of toe alignment under controlled tension allows identification of residual deforming forces and supports stepwise adjustment of the surgical strategy. This flexible, mechanism-based approach may help avoid undercorrection or unnecessary extensive dissection. The pathology-oriented framework proposed in this review provides a structured approach to this decision-making process. By linking deformity pattern to underlying biomechanics and anatomical structure, it offers a rational basis for selecting the optimal level of intervention and improving surgical outcomes.

Despite these insights, the current evidence remains limited by the predominance of case reports and small case series, with few comparative studies directly evaluating proximal and distal approaches. Further prospective and biomechanical studies are required to validate this framework and to refine criteria for surgical level selection.

This review has several limitations. First, as a narrative review, study selection was not based on predefined systematic criteria, introducing potential selection bias. Second, the available evidence is largely limited to case reports and small case series, restricting the strength of the conclusions. Third, direct comparative studies between proximal and distal surgical approaches are lacking. The current framework is derived largely from anatomical studies, biomechanical observations, case reports, and small case series. Despite these limitations, it provides a structured approach to surgical decision-making and a foundation for future research.

## Conclusions

The current evidence is limited by the predominance of case reports and small case series, with a lack of quantitative and comparative data. Further prospective studies are required to validate this framework and its clinical applicability. Despite these limitations, a pathology-oriented approach provides a rational framework for aligning surgical strategy with the dominant mechanical pathway in checkrein syndrome. Recognition of both proximal tethering and distal intertendinous coupling allows a more comprehensive understanding of deformity and its variability.

Surgical level selection should be guided by the underlying pathological mechanism. Hallux-dominant deformity is more consistent with proximal pathology, whereas multi-toe involvement associated with intertendinous coupling supports consideration of distal intervention. Careful assessment of deformity pattern and dynamic behavior remains essential for surgical planning and has the potential to improve outcomes, reduce recurrence, and avoid unnecessary proximal procedures. The proposed pathology-oriented framework should be regarded as a hypothesis-generating conceptual model rather than a clinically validated decision algorithm.

## Appendices

Appendix A 
